# Biomechanical Performance of Polyethylene Fibers and Fiber‐Reinforced Composite Resins in Direct Restorations: A Scoping Review

**DOI:** 10.1111/jerd.70012

**Published:** 2025-07-30

**Authors:** Beatriz Fernandes, Joana A. Marques, Gabriela Almeida, João Carlos Ramos, Markus B. Blatz, Rui I. Falacho

**Affiliations:** ^1^ Dentistry Department, Faculty of Medicine University of Coimbra Coimbra Portugal; ^2^ Institute of Endodontics, Faculty of Medicine University of Coimbra Coimbra Portugal; ^3^ Center for Innovation and Research in Oral Sciences (CIROS), Faculty of Medicine University of Coimbra Coimbra Portugal; ^4^ Institute of Operative Dentistry Faculty of Medicine, University of Coimbra Coimbra Portugal; ^5^ Department of Preventive and Restorative Sciences University of Pennsylvania School of Dental Medicine Philadelphia Pennsylvania USA

**Keywords:** composite resins, fiber reinforced composite, flexural strength, polyethylene fiber

## Abstract

**Objective:**

To assess the biomechanical performance of the application of polyethylene fiber and fiber‐reinforced composite resins in direct restorations.

**Materials and Methods:**

An electronic search was performed in Cochrane Library, Embase, and Pubmed on January 16th, 2025, with previously identified MeSH and free text terms by two independent authors. A total of 1127 papers were initially obtained. Exclusion criteria comprised nonrelevant materials, evaluation of pins and posts, material characterization, indirect or provisional restorations, and the use of artificial, animal, or immature teeth.

**Results:**

From the 754 studies remaining after duplicates removal, 679 were excluded after title and abstract screening, followed by the exclusion of 7 papers after full‐text assessment for eligibility. Additionally, 10 full texts were not obtained after requesting the authors. Therefore, a total of 58 studies were included in this scoping review. When compared to conventional composites, fiber‐reinforced composite resins proved to be beneficial in 82.1% of the articles, while polyethylene fibers improved the biomechanical performance in 91.7%.

**Conclusions:**

The individual application of fiber‐reinforced composite resins and polyethylene fibers demonstrates promising potential in dental direct restorations. No adverse biomechanical outcomes were reported, with findings either supporting their use or indicating no significant difference. Nevertheless, the results are ambiguous and insufficient to directly compare both materials or assess the effects of their combined use. It is recommended to establish standardized guidelines for future research to enhance the comparability of results, explore long‐term outcomes, and customize study designs to more closely resemble clinical settings.

**Clinical Significance:**

Fiber‐reinforced composite resins and polyethylene fibers have emerged in an attempt to improve the biomechanical performance of direct restorations. However, a comprehensive review of the existing literature is necessary to assess their influence on biomechanical outcomes and identify knowledge gaps, thereby guiding future research.

## 1 Introduction

The loss of dental structure results in a significant impairment of the physical and mechanical properties of the tooth, influenced by several factors such as the number of remaining walls, marginal ridge loss, cavity configuration, extent of occlusal isthmus, and depth of the preparation [1, 2]. Each one of these factors, especially when combined, can lead to greater cusp deflection, rendering teeth more prone to fracture [1]. This scenario is particularly aggravated for endodontically treated teeth due to the effects of root canal irrigants and intracanal medication, impaired proprioception, and the additional removal of dentin [3]. In an attempt to restore the tooth's original integrity, numerous materials and techniques have been developed and investigated.

Conventional composite resins (CRs) are currently the most commonly used restorative material, offering satisfactory mechanical properties, esthetics, and clinical performance [4]. However, the direct restoration of large cavities has shown some limitations, particularly in high‐load bearing areas [5]. These limitations comprise a notable lack of toughness and polymerization contraction, which can lead to restoration failure through micro‐leakage, secondary caries, or fracture of the remaining cavity walls [6, 7]. Hence, it has become imperative to optimize this material or identify alternatives capable of overcoming its deficiencies.

The application of fibers emerged in the field of dentistry over 30 years ago, with fiber‐reinforced composites ranging in applicability across prosthodontics, orthodontics, periodontology, and pediatric dentistry [8, 9]. These materials present promising characteristics, and multiple fibers have been used, such as carbon, aramid, polyethylene, and glass [8].

Fiber‐reinforced composites consist of three major components: the matrix (continuous phase), the fibers (dispersed phase), and the interphase region (matrix/fibers interphase) [8]. It is speculated that when a load is applied to the polymer matrix, the stress is transferred to the fibers, which act as stress dissipators [4]. Therefore, the mechanical performance is influenced by the type of composite resin used, the quantity of fibers in the resin matrix, the length, form, and orientation of these fibers, their adhesion to the polymer matrix, and the resin impregnation [10, 11].

EverX Posterior (GC Europe N.V., Leuven, Belgium) is the most studied short‐fiber‐reinforced composite (SFRC). Its composition includes a combination of short E‐type glass fibers and barium glass particles that are randomly oriented within the polymer matrix, thereby providing omnidirectional reinforcement [4, 6].

In addition to fiber‐reinforced composite resins, the use of polyethylene fibers has also been introduced as an attempt to reinforce the remaining tooth structure, consisting of aligned polymer chains with a low modulus of elasticity [12]. One of the most widely used polyethylene products is Ribbond (Ribbond Inc., Seattle, WA, USA), a reinforced ribbon made of ultra‐high molecular weight polyethylene fiber with a high elastic modulus. This material undergoes a cold gas plasma treatment to enhance its adhesion to restorative materials. The goal is to create a stress‐absorbing layer that acts as an internal splint, although it provides only bidirectional reinforcement [6, 13].

Given the heterogeneity of materials and techniques, a scoping review approach was chosen to map, report, and discuss the available literature on the topic, with the ultimate purpose of identifying knowledge gaps [14]. Thus, the aim of this scoping review is to assess the biomechanical performance of fiber‐reinforced resins and polyethylene fibers in direct restorations.

## 2 Methodology

To conduct this scoping review, the Preferred Reporting Items for Systematic Reviews and Meta‐Analyses extension for Scoping Reviews (PRISMA‐ScR) were followed, as well as the methodological guidelines and discipline‐specific recommendations to ensure transparent and rigorous reporting published by Prott et al. [15, 16] In order to establish the search strategy, the following question was formulated: “Does the use of polyethylene fibers and fiber‐reinforced resins affect the biomechanical performance of direct restorations?”

## 3 Eligibility Criteria

Regarding study selection, the inclusion criteria comprised English‐written in vitro and clinical studies that evaluated the mechanical performance of SFRCs or polyethylene fibers. The exclusion criteria were the following: (1) nonrelevant materials, such as glass fibers; (2) assessment of posts or dentin pins properties; (3) materials characterization; (4) indirect or provisional restorations; and (5) studies using immature, animal, or artificial teeth.

## 4 Information sources and search strategy

An electronic search was carried out on PubMed, Cochrane, and Embase, without chronologic restrictions up to the day it was performed: January 16th, 2025. The following MeSH/Emtree Terms were previously identified: “flexural strength” and “composite resins,” and Free text terms were used and combined to define the search key, as displayed in Table 1.

**TABLE 1:** Search strategy.

Search terms	#1	“Flexural resistance” OR “fracture resistance”
	#2	“Composite resin”
	#3	“Polyethylene fiber” OR “polyethylene fiber” OR “fiber reinforcement” OR “fiber reinforcement” OR “fiber‐reinforced restoration” OR “fiber‐reinforced restoration” OR “ribbond” OR “fiber‐reinforced composite” OR “fiber‐reinforced composite” OR “fiber‐reinforced composite restoration” OR “fiber‐reinforced composite restoration” OR “fiber‐reinforced resins” OR “fiber‐reinforced resins” OR “short fiber‐reinforced composite” OR “short fiber‐reinforced composite” OR “short fiber composite” OR “short fiber composite” OR “ever x posterior” OR “ever x flow”
MeSH terms/emtree terms	#4	Flexural strength
	#5	Composite resins
Search key	(#1 OR #4) AND (#2 OR #5) AND #3	

## 5 Selection of sources of evidence

Duplicate removal was conducted resorting to Zotero reference manager. Subsequently, titles and abstracts were independently screened by two researchers. In case of disagreement, a third researcher was involved to reach a final decision. The selected articles were then subjected to a comprehensive full‐text analysis and eligibility assessment, considering the established inclusion and exclusion criteria. A manual search was conducted through the reference list of all included studies to identify additional relevant papers.

## 6 Data extraction

Data were extracted regarding sample size and teeth type, endodontic treatment, cavity configuration, control group, experimental groups, compared materials, application techniques, tested parameters, and main conclusions. The papers were grouped by studied materials to facilitate comparisons.

## 7 Results

**FIGURE 1:**
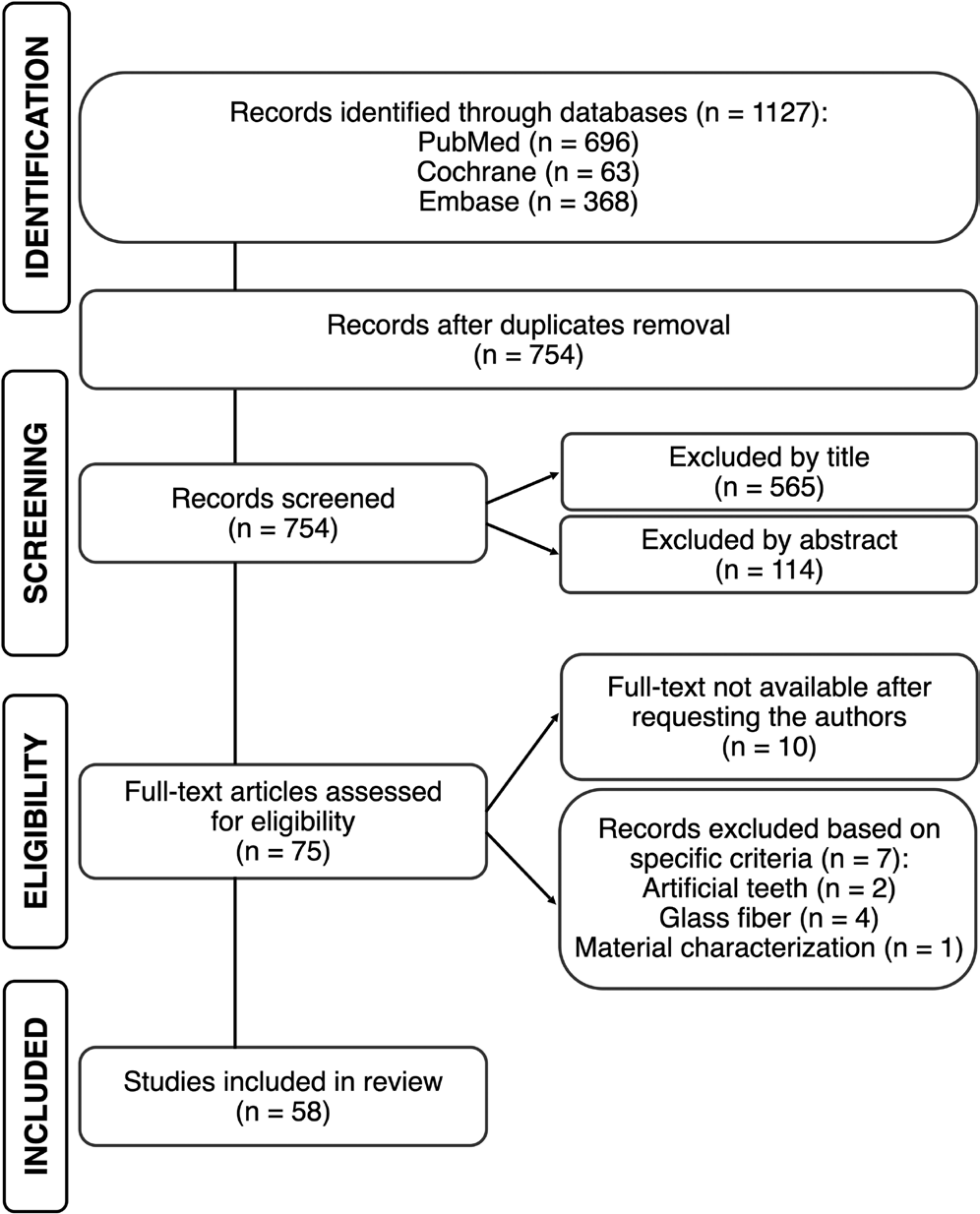
Flowchart of study selection.

The study selection process flowchart is depicted in Figure 1.

A total of 1127 studies were collected from all the databases through an electronic search, with 696 publications identified in PubMed, 63 in Cochrane, and 368 in Embase. After duplicate removal, 754 papers remained. Data screening based on titles and abstracts resulted in the exclusion of 679 articles, leaving 75 articles for full‐text assessment. Of these, 7 were excluded due to specific criteria—two studies employed artificial teeth, four assessed exclusively glass fiber, and one focused solely on the characterization of the materials under investigation—and 10 could not be retrieved after contacting the authors. Hence, 58 studies were included in this review. The methodological data and main conclusions of the included studies evaluating SFRC, polyethylene fiber, or both are outlined in Tables 2, 3, 4, respectively.

**TABLE 2:** Data extraction of the included studies evaluating SFRC.

Authors	Sample	Cavity configuration	Root canal treatment	Control groups	Experimental groups		Test/outcome measure	Main conclusions
					Compared materials	Restorative technique		
Nezir et al. [17]	100 mandibular first molars	Conservative MO access cavity	Yes	Intact teeth; Unrestored teeth	Filtek One Bulk Fill Restorative; EverX Posterior; Cytec Blanco	Single 4 mm increment followed by a 4 mm thick bulk fill resin composite layer	Thermocycling procedure Fracture resistance (45° at the central fossa) Failure Mode	EverX posterior significantly increased the fracture resistance of endodontically treated first mandibular molars.
Abdel‐Maksoud et al. [18]	60 maxillary premolars	MOD + Access cavity	Yes	Intact teeth; Unrestored teeth	VisCalor bulk; X‐tra fil; EverX flow; Charmfil Plus; Grandioso flow	Bulk fill technique	Thermocycling procedure Fracture resistance 0°	Short‐fiber reinforced composite demonstrated significantly higher fracture resistance compared to other types of composites assessed in this study.
Hazar and Hazar [19]	90 double‐rooted premolars	MOD + Access cavity	Yes	Intact teeth	G‐aenial universal injectable; EverX Flow; EverStick C&B	Proximal walls built with composite, followed by the application of a 4 mm layer of SFRC to the core and a final layer of composite.	Fracture Resistance 30° Failure mode	Direct restorations using unidirectional long fibers with modified transfixed technique or flowable SFRCs increase the fracture strength of endodontic treated premolars.
Magne and Milani [20]	36 third molars	MOD	No	Conventional composite	Grandia direct; EverX Flow; Cerasmart 270	Layering technique with 7 increments, proximal walls and final occlusal layer filled with conventional composite	Accelerated fatigue test Enamel crack tracking Failure mode (30°)	The restorability and fracture resistance of teeth when using SFRC increases, especially when an inlay is applied. The opposite happens with shrinkage‐induced cracks which decrease as SFRC and inlays are used.
Garoushi et al. [21]	120 mandibular molars	MOD + lingual cusp removed	No	—	G‐aenial Posterior; Cerasmart 270; LiSi; EverX Flow	A core of SFRC, varying in thickness from 1 to 5 mm, with or without restoration, either direct or indirect.	Cyclic fatigue aging Fracture Load Test (0°) Failure type	Direct restorations using SFRC either alone or as a core in combination with conventional composites show higher fracture resistance, as well as a reduction of the incidence of catastrophic fractures compared to restorations without SFRC was observed.
Naik et al. [22]	84 maxillary premolars	MOD + Access cavity	Yes	Intact teeth	Filtek Z250XT; FRC Postec Plus; IPS E‐max CAD; EverX Posterior	Incremental technique followed by occlusal layer with conventional composite; Same technique as previously mentioned + application of a horizontal post	Fracture resistance test (0°) Failure Mode	The integration of a horizontal post into the SFRC and conventional composite restoration lead to an increase in fracture resistance, which was also observed in teeth restores with ceramic inlays. However, no significant difference was observed between conventional composite and the fiber reinforced restorations. All restored teeth showed mostly favorable fractures.
Selvaraj and Krithikadatta [23]	60 maxillary first premolars	MOD + Access cavity	Yes	Intact teeth; Unrestored teeth	SDR Flow +; EverX Posterior	Incremental technique followed by occlusal layer with conventional composite (1 mm)	Thermocycling Fracture resistance test (0°) Failure Mode	SFRC demonstrated increased fracture resistance compared do SDR in endodontically treated teeth in high stress bearing zones, as well as a higher number of favorable fractures.
Battancs et al. [24]	72 mandibular molars	Class I occlusal cavity	No	—	Filtek ultimate; EverX Posterior; Surefil SDR	Single increment following the dentin anatomy + occlusal layer with conventional composite	Fracture resistance (0°) Failure mode Microleakage testing and gap formation evaluation	Bulk‐fill showed higher fracture resistance compared to conventional composite but no difference to SFRC and better internal adaptation to the cavity walls. SFRC showed more restorable fracture behavior.
Fráter et al. [25]	180 maxillary premolars	MOD + Access cavity	Yes	Conventional composite	G‐aenial Universal injectable; EverStick Post; Cerasmart 270; EverX Flow	Direct post and core with flowable SFRC using the Bioblock technique + occlusal layer with conventional composite; flowable SFRC used as luting and core building material	Accelerated fatigue test (0°) Failure type	Custom‐made fiber post and SFRC as post luting core material, with or without cuspal coverage, performed well in terms of fatigue resistance and survival when used for the restoration of endodontically treated premolars. Indirect overlay was superior to direct overlay. All restored groups showed mostly nonrestorable fractures.
Lakshmi Durga et al. [26]	50 mandibular premolars	Resected crowns + access cavity	Yes	Intact teeth	Te‐Econom Plus; Tetric‐N‐Ceram; Multicore flow; EverX Posterior	Not specified	Fracture resistance test (0°)	Reinforcement with SFRC showed significantly higher fracture resistance compared to nano‐hybrid and dual‐cure composite resins.
Molnár et al. [27]	250 mandibular third molars	MO + Access cavity	Yes	Conventional composite	G‐aenial Posterior; Fugi II LC; Equia Forte; EverX Posterior; EverX Flow	Horizontal incremental technique followed by occlusal layer with conventional composite	Cyclic loading Fatigue resistance test (0°) Failure mode with SEM images	The use of SFRC as dentin replacing material increased the fracture resistance. All groups showed mostly catastrophic fractures but a change of the direction of the fracture upon contact with the SFRC layer.
Fráter et al. [28]	170 maxillary premolars	MO + Access cavity	Yes	Intact teeth	G‐aenial posterior; Everstick post; EverX Posterior; EverX Flow	Bulk fill technique + occlusal layer with conventional composite; Flowable SFRC used to lute posts; Individually made post with SFRC	Accelerated fatigue test (135°) Failure type Microgap determination test Microhardness test	Restorations with the use of individually‐made post with fiber reinforced composite post and luted with flowable SFRC, showed a promising achievement regarding fatigue resistance and survival. All restored groups presented mostly irreparable fractures.
Kaur et al. [29]	90 maxillary premolars	MOD + Access cavity	Yes	Intact teeth; Unrestored teeth	Filtek P60; Tetric‐N‐Ceram Bulk Fill; Luxacore Z Dual; EverX Posterior	Incremental technique up to the occlusal level	Fracture resistance test (0°)	EverX posterior achieved higher fracture resistance than all restored groups, being considered a desirable material to restore endodontically treated teeth, providing long term survival.
Kale et al. [30]	44 maxillary premolars	MOD	No	Intact teeth	Beautifill II LS; Prevest Fusion Universal; EverX Posterior	Proximal walls built with composite, followed by the application of the SFRC to the core.	Thermocycling Fracture resistance test (0°)	It was observed that the nanohybrid composite with Giomer chemistry provided the highest reinforcement of weakened maxillary bicuspids, followed by SFRC.
Shilpa‐Jain et al. [31]	60 mandibular premolars	Cervical lesion centered access cavity design (CLCAC); Traditional access cavity	Yes	Intact teeth; CLCAC without restoration	Tetric‐N‐Ceram; Everstick; EverX Posterior	Incremental technique followed by occlusal layer with conventional composite	Thermocycling Cyclic Compressive load Fracture resistance Failure mode (−)	Application of SFRC improved the fracture resistance. Favorable fractures occurred when CLCAC were restored with fiber reinforced composites.
Frankenberger et al. [32]	120 mandibular third molars	MOD + Access cavity	Yes	Intact teeth	Tetric EvoCeram Bulk Fill; EverX Flow; Essentia Universal; Emax CAD PC/FC; Celtra Duo PC/FC; Cercon ht.; Degunorm	Incremental oblique technique followed by occlusal layer with conventional composite	Thermomechanical loading Failure mode (−)	In the groups where direct restorations were performed, no statistically significant differences were observed between the conventional composite and the SFRC groups. Catastrophic fractures were noticed in all groups.
Bijelic‐Donova et al. [33]	60 maxillary third molars	MOD + Access cavity	Yes	Intact teeth	G‐aenial Posterior; EverX Posterior	Incremental technique followed by occlusal layer with conventional composite	Chewing test Fracture resistance (0°) Failure mode	Fracture resistance of teeth restored with SFRC was significantly higher than intact teeth restored with conventional composite resin, and prevented the fracture line propagation towards the CEJ, having significantly more restorable fractures than intact teeth and direct restorations.
Hartanto et al. [34]	30 maxillary premolars	Class II box only	No	Conventional composite	Filtek Z250XT; EverX Posterior	Bulk fill technique followed by occlusal layer with conventional composite	Thermocycling Fracture Resistance (13,5°)	The use of SFRC as intermediate layer enhanced the fracture resistance of class II composite restoration, compared to nanohybrid composite.
de Kuijper et al. [35]	150 third molars	Decoronated teeth	Yes	Endodontic access cavity only	Clearfil AP‐X; GC Essentia; RelyX fiber post; Tetric EvoFlow; Clearfil DC core plus dentin; EverX Posterior	Proximal walls raised with conventional composite + incremental technique with SFRC + occlusal layer of conventional composite	Thermocycling Chewing simulator Fracture Resistance (0°) Failure Mode	The application of SFRC did not demonstrate improvements in terms of fracture resistance when compared to the other groups, namely the conventional composite group. On the other hand, its utilization was associated with more repairable fractures.
Shafiei et al. [36]	120 maxillary premolars	MOD	No	Conventional composite; SFRC	Filtek Z250; EverX Posterior	Incremental technique followed by occlusal layer with conventional composite	Fracture resistance test (0°) Failure Mode	While SFRC exhibited greater fracture resistance compared to conventional composite resin, the various liners did not demonstrate any significant influence on it.
Eapen et al. [37]	60 maxillary premolars	MOD + Access cavity	Yes	Intact teeth; Unrestored teeth	Filtek P60; Interlig Angelus; EverX Posterior	Incremental technique followed by occlusal layer with conventional composite	Fracture resistance test (0°) Failure Mode	SFRC in MOD cavities increased the fracture resistance of endodontically treated premolars. Concerning the type of fracture, in SFRC restorations all fractures occurred at the enamel level, similar to intact teeth.
Barreto et al. [38]	32 mandibular premolars	MOD + lingual cusp removed	Yes	Conventional composite	G‐aenial posterior; EverX Posterior	Proximal walls raised with conventional composite +4 mm bulk increment of SFRC + occlusal layer of conventional composite	Fatigue resistance test Fracture resistance test (0°; 45°) Failure mode analysis using SEM Stress distribution‐FEA	Fatigue resistance and fracture resistance did not increase with the application of SFRC. Fractures were mostly repairable when tests were conducted at a 0° angle, but mostly unrepairable at a 45° angle. The areas with the highest stress were found at the buccal DEJ region.
Atalay et al. [39]	72 maxillary premolars	MOD + access cavity	Yes	Intact teeth; Unrestored teeth	Filtek Bulkfill; Surefil SDR Flow; Ceram.X Mono; G‐aenial Posterior; Tetric‐N‐Ceram; EverX‐Posterior	A 4 mm layer of SFRC was applied and the occlusal layer restored with conventional composite	Thermocycling Fracture resistance (perpendicular to the long axis of the tooth) Failure Mode	There were no statistically significant differences among the experimental groups, indicating that the application of SFRC did not prove to be advantageous.
Fráter et al. [40]	130 mandibular third molars	MOD	No	Intact teeth	G‐aenial posterior; EverX posterior	Horizontal incremental technique (2 mm thick layers) + occlusal layer of conventional composite; Oblique incremental technique (2 mm thick layers) + occlusal layer of conventional composite	Fracture resistance test (0°) Failure Mode	Although the results were not statistically significant, there is a tendency towards higher fracture resistance SFRC with an oblique layering technique. Only the SFRC in oblique increments yield restorable fractures.

**TABLE 3:** Data extraction of the included studies evaluating polyethylene fiber.

Authors	Sample	Cavity configuration	Root canal treatment	Control groups	Experimental groups		Test/outcome measure	Main conclusions
					Compared materials	Fibers application technique		
AlJarboua et al. [41]	54 maxillary premolars	MOD/MOD + Access cavity	Yes	Intact teeth	Filtek Z250 XT; Ribbond; Tetric N‐Ceram	Pulpal floor; Proximal walls; Pulpal floor and proximal walls	Fracture resistance (0°) Fracture type Failure mode	Ribbond exhibited higher fracture resistance than conventional restorations in compromised upper premolars.
Verma et al. [42]	80 mandibular premolars	MOD + Access cavity	Yes	Intact teeth; Unrestored teeth	Filtek P60; Cention N; Ribbond; Fiber post	Bucco‐lingual in a groove on the occlusal surface	Fracture resistance (45°)	Fiber reinforcement significantly increased the fracture resistance of endodontically treated premolars.
Shafiei et al. [43]	80 maxillary premolars	MOD + Access cavity	Yes	Intact teeth; Unrestored teeth	Tetric‐N‐Ceram; TN Bulk Fill; X‐tras base; Ribbond	Buccal, lingual and pulpal walls	Thermocycling Fracture resistance test (0°) Failure Mode	Fiber application increased the fracture resistance and reduced the number of unfavorable fractures. Flowable bulk fill reached the strength level of conventional composite with fiber.
Eliguzeloglu Dalkılıç et al. [44]	80 mandibular premolars	MO + Access cavity	Yes	Intact teeth; Intact teeth subjected to thermomechanical aging	Estelite bulk fill flow; Ribbond	Buccal, lingual and pulpal walls; Buccal, lingual and pulpal walls + between the 2 layers of composite	Thermomechanical aging Fracture resistance test (0°) Failure Mode	Polyethylene fiber application beneath the bulk fill composites did not improve the fracture resistance. On the other hand, the type of fracture proved to be more favorable with its application.
Galyan et al. [45]	60 maxillary central incisors	Oblique fracture + palatal slot	No	Intact teeth	Filtek Z350; Ribbond	Applied into the palatal slot, protruding from the fractured margin. One group with one slot and other with two.	Fracture resistance (45°)	Use of polyethylene provided strength almost equivalent to natural teeth (1 slot).
Aslan et al. [46]	105 single‐rooted mandibular premolars	MOD + Access cavity	Yes	Intact teeth; Unrestored teeth	Filtek Ultimate; Ribbond; RelyX Fiber post	Bucco‐lingual in a groove on the occlusal surface	Fracture resistance test (45°) Failure Mode	The application of occlusal Ribbond and horizontal post increased the fracture resistance of endodontically treated premolars with MOD cavities. No differences were found between the experimental groups regarding the failure mode.
Hshad et al. [47]	48 mandibular premolars	MOD + Access cavity	Yes	Intact teeth	Clearfil Majesty Flowable universal composite; Clearfil AP_X Composite; Ribbond	Buccal, lingual and pulpal walls	Fracture resistance test (0°) Failure Mode	Ribbond had similar fracture resistance to intact teeth. Flowable resin did prove advantageous. Concerning the failure mode, polyethylene group exhibited the most favorable outcomes, with fractures occurring mostly on enamel.
Khan et al. [48]	140 first molars	MOD + Access cavity	Yes	Intact teeth; Unrestored teeth	Te‐econom Plus; Ribbond; Everstick C&B; Dentapreg UFM; Bioctris Fiber	Buccal, lingual and pulpal walls (Everstick C&B‐ 2 strips applied adjacent to each other)	Fracture resistance test (0°)	Restorations with fibers significantly increased the fracture resistance of the restored teeth. Among the fibers Everstick and Bioctris showed better results. E‐glass system is able to better reinforce restored teeth than S2 glass and polyethylene fibers
Miao et al. [12]	50 maxillary premolars	MOD with palatal cusp removed and buccal cusp reduced	Yes	Intact teeth	Filtek P60; Rely X fiber post; Ribbond	Buccal to lingual over the cusps, covered with 2 mm of composite	Thermocycling Fracture resistance test (0°) Failure Mode	Regardless of the use of the fiber posts, restorations with polyethylene fiber provided superior fracture resistance of premolars with defective palatal cusps and endodontic treatment compared with conventional direct restorative techniques.
Rahman et al. [49]	40 maxillary premolars	MOD + Access cavity	Yes	Conventional composite	Filtek Z250; Ribbond	Bucco‐lingual in a groove on the occlusal surface; Buccal, lingual and pulpal walls; Both combined	Thermocycling Fracture resistance test (0°) Failure Mode	When both techniques were combined, the fracture resistance increased but most of the favorable fractures were observed when the fiber was applied under the restoration
Shafiei et al. [50]	96 maxillary premolars	MOD + Access cavity	Yes	Intact teeth; Unrestored teeth	Z250; Filtek P90; Ketac N 100; Ribbond	Buccal, lingual and pulpal walls	Thermocycling Fracture resistance test (0°) Failure mode	The fiber insertion revealed no additional positive effect on the fracture resistance of restored teeth using methacrylate and silorane based composites, however increased the number of restorable fractures when methacrylate was used.
Kalburge et al. [51]	120 maxillary premolars	MOD and DO + Access cavity	Yes	Intact teeth; Unrestored teeth	Filtek Z‐100; Ribbond	Buccal, lingual and pulpal walls	Thermocycling Fracture resistance test (45°)	Inserting polyethylene fiber in Class II DO and MOD preparations significantly increased fracture resistance.
Luthria et al. [52]	50 maxillary premolars	MOD + Access cavity	Yes	Intact teeth; Unrestored teeth	Filtek Z350 XT; Interlig; Ribbond	Bucco‐lingual in a groove on the occlusal surface	Thermocycling Fracture Resistance (0°)	Glass fiber reinforced group showed higher fracture resistance values than the other experimental groups, however the difference is not statistically significant.
Oskoee et al. [53]	45 single rooted maxillary premolars	MOD + Access cavity	Yes	—	Filtek Z250; Interlig; NSI	Buccal, lingual and pulpal walls	Thermocycling Fracture resistance test (0°) Failure Mode	Glass fiber obtained significant higher fracture resistance results compared to polyethylene fiber and composite. The fractures were mostly unfavorable in all groups.
Badakar et al. [54]	40 maxillary central incisors	Incisal edge cut obliquely	No	Intact teeth	Esthet X; Ceram X; Ribbond	Placed into a palatal cavity, extending it above the fracture edge	Fracture resistance (45°) Failure mode (FM)	Fiber reinforcement of the conventional composites has recovered the fracture resistance of the restoration comparable to the natural tooth.
Ayad et al. [55]	50 mandibular molars	Class I and II cavities	No	Intact teeth	Prodigy; Ribbond	Buccal to lingual at the area of the marginal ridge	Fracture resistance test (0°)	Fiber‐reinforced composites tested improved the fracture resistance of Class I cavities.
Cobankara et al. [56]	60 mandibular molars	MOD + Access cavity	Yes	Intact teeth; Unrestored teeth	Cavez Avalloy‐II spill; Clearfil photoposterior; Ribbond; Hybrid ceramic material	Buccal, lingual and pulpal walls	Fracture resistance test (0°) Failure Mode	Fiber reinforcement of the conventional composites has recovered the fracture resistance of the restoration comparable to the natural tooth.
Sengun et al. [57]	80 mandibular premolars	MOD + Access cavity	Yes	Intact teeth; Unrestored teeth	Clearfil AP‐X; Ribbond	Bucco‐lingual in a groove on the occlusal surface	Fracture Resistance (45°) Failure mode	Inserting polyethylene fiber buccolingually on the occlusal surface in endodontically treated premolars with MOD cavities increased the fracture resistance and prevented unfavorable fractures.
Belli et al. [58]	50 mandibular second molars	MOD + Access cavity	Yes	Intact teeth; Unrestored teeth	Clearfil AP‐X; Protect liner F; Ribbond	Applied bucco‐lingual in a groove on the occlusal surface; Buccal, lingual and pulpal walls	Fracture resistance test (0°) Failure Mode	Composite restoration increased the fracture strength of the studied teeth. Polyethylene fiber before and after the restoration significantly increased the fracture resistance, when placed in the occlusal surface of the restoration from buccal to lingual direction higher resistance was observed.
Belli et al. [59]	60 mandibular molars	MOD + Access cavity	Yes	Intact teeth; Unrestored teeth	Clearfil AP‐X; Protect Liner F; Ribbond	Buccal, lingual and pulpal walls	Fracture resistance (0°)	Inserting polyethylene fiber in endodontically treated teeth with MOD cavities significantly increased fracture strength.

**TABLE 4:** Data extraction of the included studies comparing SFRC application and polyethylene fiber.

Authors	Sample	Cavity configuration	Root canal treatment	Control groups	Experimental groups		Test/outcome measure	Main conclusions
					Compared materials	SFRC and fibers application technique		
Negm^a^ et al. [60]	32 M	Class I	No	Conventional composite	Ribbond; EverX Posterior; EverX Flow; Tetric N Flow	SFRC applied resorting to a bulk‐fill technique. Ribbond application not specified.	Thermocycling procedure Fracture Resistance 0° Failure type FEA and SEM	Teeth restored with fiber‐reinforced composite had the highest fracture resistance, followed by fiber‐reinforced flowable resin composite. Teeth restored with the ultra‐polyethylene fiber, followed by the flowable bulk‐fill composite had the lowest resistance.
Ramírez‐Gómez et al. [61]	100 maxillary premolars	MOD + Access cavity; Occluso‐buccal (OB) + Access cavity	Yes	MOD without fibers; OB without fibers	IPS Empress Direct; Clearfill AP‐X esthetics flow; Fuji II LC; Ribbond; EverX Posterior	Axial walls restored with conventional composite. All cavities restored with SFRC following an incremental technique, being the final layer with conventional composite. All fibers applied into de pulp chamber floor except the circumferential ones Ribbond: mesial to distal; Buccal to palatal; mesial to distal + buccal to palatal (2 pieces of fiber); circumferential	Fracture resistance (30°) Failure Mode Failure Type	Location and orientation of the fibers did not affect the fracture resistance. All the fiber positions minimized unrestorable fractures. Multidirectional fibers decreased the fracture resistance compared to unidirectional.
Tsertsidou et al. [62]	60 mandibular molars	MOD	No	Conventional composite	Tetric; Ribbond; EverX Posterior; Brilliant Crios	Ribbond: mesial, distal, internal and occlusal walls built with conventional composite, the Ribbond pieces (3x3) were pressed against the interior tooth surfaces SFRC: A 4 mm layer of SFRC was applied and the occlusal layer restored with conventional composite	Thermocycling procedure Fracture Resistance test (0°) Fracture type	CAD/CAM and fiber reinforced techniques in MOD cavities showed improvements in fracture resistance when compared to composite resin alone. SFRC demonstrated most favorable outcomes in terms of fracture resistance compared to other approaches.
Volom et al. [63]	120 mandibular molars	MOD + Access cavity	Yes	SFRC	G‐aenial Posterior; G‐aenial Universal Injectable A3; Fibrekleer post; Ribbond; EverX flow (bulk and dentin shade)	Axial walls restored with conventional composite SFRC: bulk technique leaving 2 mm for the flowable dentin shade, with cuspal coverage using conventional composite Ribbond: Transcoronal splint	Accelerated fatigue testing protocol (0°) Failure Mode	Transcoronal splint with direct cuspal coverage presented higher survival rate, contributing to the strengthening of the restoration. However, the same does not seem to apply when flowable SFRC is used.
Abdulamir and Majeed [64]	40 maxillary premolars with 2 roots	MOD + Access cavity	Yes	Conventional composite	G‐aenial A'chord; GC fiber post; Ribbond; EverX Posterior	Ribbond: buccal, lingual and pulpal walls; applied circumferentially inside the cavity walls SFRC: proximal walls raised with conventional composite followed by bulk application of SFRC, restoring the occlusal layer with conventional composite	Fracture resistance test (0°) Failure Mode	Incorporating Ribbond fibers within composite restoration, whether as a wallpapering or on the floor, significantly increased the fracture resistance of endodontically treated teeth.
Soto‐Cadena et al. [65]	40 maxillary premolars	MOD + Access cavity	Yes	Intact teeth	Clearfill AP‐X Esthetics Flow; Ribbond; EverX Posterior	Ribbond: buccal, lingual and pulpal walls SFRC: EverX applied, restoring the occlusal layer with conventional composite using an incremental technique Ribbond + SFRC: Ribbond applied as described above then restored with SFRC with the same technique above	Fracture resistance test (30°) Failure type Failure Mode	The use of PRF and SFRC individually was beneficial to the fracture patterns. PRF + SFRC increased the fracture resistance values.
Deger et al. [66]	32 maxillary premolars	MOD	Yes	Conventional composite	Filtek Z250; Estelite Bulk Fill Flow; Ribbond; NovaPro Flow	Ribbond: Pulpal wall SFRC: Used as a liner (1 mm thick)	Gap formation Cuspal deflection Fracture strength (0°) Failure mode	Different intermediary layers did not affect cuspal deflection and fracture strength. Polyethylene exhibit greater gap formation when compared with other tested groups.
Albar and Khayat [67]	72 M	MOD	No	Conventional composite	Filtek Z250; Activa Bioactive; Ribbond; EverX Posterior	Ribbond: Buccal, lingual and pulpal walls SFRC: EverX applied, restoring the occlusal layer with conventional composite Ribbond + SFRC: Ribbond applied into the buccal, lingual and pulpal walls followed by EverX application, restoring the occlusal layer with conventional composite	Thermocycling Fracture resistance (0°) Scanning electron microscope (SEM)	A higher fracture resistance can be achieved when reinforcing nanohybrid composite with EverX Posterior or Ribbond individually.
Sáry et al. [68]	240 mandibular third molars	MOD	No	Intact teeth	G‐aenial Posterior; Everstick net; Ribbond; EverX Posterior	Proximal walls were made with conventional composite Ribbond: buccal, lingual and pulpal walls; applied bucco‐lingual in a groove on the occlusal surface; applied circumferentially inside the cavity walls; transcoronal splinting SFRC: bulk application of SFRC, restoring the occlusal layer with conventional composite	Fracture resistance test (0°) Failure Mode	The use of polyethylene fiber seems to be always beneficial, regardless the position. Glass fiber with SFRC is highly dependent on their position. Bulk applied SFRC can be used to reinforce MOD cavities. Transcoronal splinting with Ribbond yield the highest fracture resistance, being even slightly higher than intact teeth.
Tekçe et al. [69]	70 mandibular first molars	Inlay cavity	Yes	Intact teeth; Unrestored teeth	G‐aenial Posterior; G‐aenial universal Flo Flowable; Surefill SDR; Ribbond; EverX Posterior	Ribbond: buccal, lingual and pulpal walls; SFRC: bulk application of SFRC, restoring the occlusal layer with conventional composite	Thermocycling Fracture resistance (0°) Failure Mode	Reinforcement of the restorations either with SFRC or polyethylene fiber modestly increased the fracture strength, but not to the intact teeth level.
Garlapati et al. [70]	50 mandibular molars	MOD	Yes	Intact teeth; Unrestored or temporary filling applied	Te‐Econom Plus; Ribbond; EverX Posterior	Ribbond: buccal, lingual and pulpal walls SFRC: applied resorting to an incremental technique, restoring the occlusal layer with conventional composite	Thermocycling Fracture resistance (0°) Failure mode	Among the tested materials EverX Posterior showed a superior fracture resistance
Ozsevik et al. [71]	50 mandibular molars	MOD + Access cavity	Yes	Intact teeth; Endodontically treated teeth with glass ionomer cement on the pulp chamber	G‐aenial Posterior; Ribbond; EverX Posterior	Ribbond: buccal, lingual and pulpal walls; SFRC: proximal walls raised with conventional composite followed by the application of SFRC, restoring the occlusal layer with conventional composite	Thermocycling Fracture resistance test (0°)	EverX Posterior under the composite restoration resulted in fracture resistance similar no intact teeth.
Gürel et al. [72]	48 maxillary premolars	MOD + palatal cusp removed	Yes	—	G‐aenial Posterior; Ribbond Thin; EverX Posterior	Ribbond: Individual post made with Ribbond and dual cure resin—a 26 mm long fiber is inserted into the canal in a U shape, with the free ends twisted within the canal SFRC: cavity and occlusal layer restored with SFRC; SFRC as core material, restoration with conventional composite; Restoration with conventional composite only	Fracture resistance (0°) Failure Mode	Application of SFRC yield not significantly but better fracture thresholds than the other studied materials.
Kemaloglu et al. [73]	48 mandibular premolars	MOD + Access cavity	Yes	—	Filtek Z550; Filtek Bulk Fill; Ribbond; EverX Posterior	Ribbond: buccal, lingual and pulpal walls SFRC: bulk application of SFRC, restoring the occlusal layer with conventional composite	Thermocycling Fracture resistance (0°) Failure Mode	Fiber reinforcement increased the fracture resistance and improved the failure mode compared to bulk fill and nanohybrid resin composites.

^a^ In addition to the data presented in the table, the identified article includes experimental groups involving deciduous teeth, which were not considered in the present review.

Of the 58 studies, 59% used premolars [12, 18, 19, 22, 23, 25, 26, 28, 29, 30, 31, 34, 36, 37, 38, 39, 41, 42, 43, 44, 46, 47, 49, 50, 51, 52, 53, 57, 61, 64, 65, 66, 72, 73], 38% used molars [17, 20, 21, 24, 27, 32, 33, 35, 40, 48, 55, 56, 58, 59, 60, 62, 63, 67, 68, 69, 70, 71] and 3% used central incisors [45, 54]. Forty‐three (43) of the studies performed root canal treatment [12, 17, 18, 19, 22, 23, 25, 26, 27, 28, 29, 31, 32, 33, 35, 37, 38, 39, 41, 42, 43, 44, 46, 47, 48, 49, 50, 51, 52, 53, 56, 57, 58, 59, 61, 63, 64, 65, 69, 70, 71, 72, 73], while 15 chose not to [20, 21, 24, 30, 34, 36, 40, 45, 54, 55, 60, 62, 66, 67, 68]. Various cavity patterns were used among the studies, with the predominant cavity type being the mesio‐occluso‐distal (MOD), used in 41 papers [18, 19, 20, 22, 23, 25, 29, 30, 32, 33, 36, 37, 39, 40, 41, 42, 43, 46, 47, 48, 49, 50, 51, 52, 53, 56, 57, 58, 59, 61, 62, 63, 64, 65, 66, 67, 68, 69, 70, 71, 73]. Additionally, 9 other variations of tooth preparation were recorded: MOD with lingual cusp removed [21, 38, 72] and a variation where the buccal cusp is reduced [12], class II box only [27, 28, 34, 44, 51, 55], access cavity and cervical lesion centered access cavity design [31], conservative mesio‐occlusal endodontic access cavity [17], decoronated teeth [26, 35], class I [24, 55, 60, 69], occluso‐buccal cavity [61] and incisal edge cut obliquely [45, 54].

**FIGURE 2:**
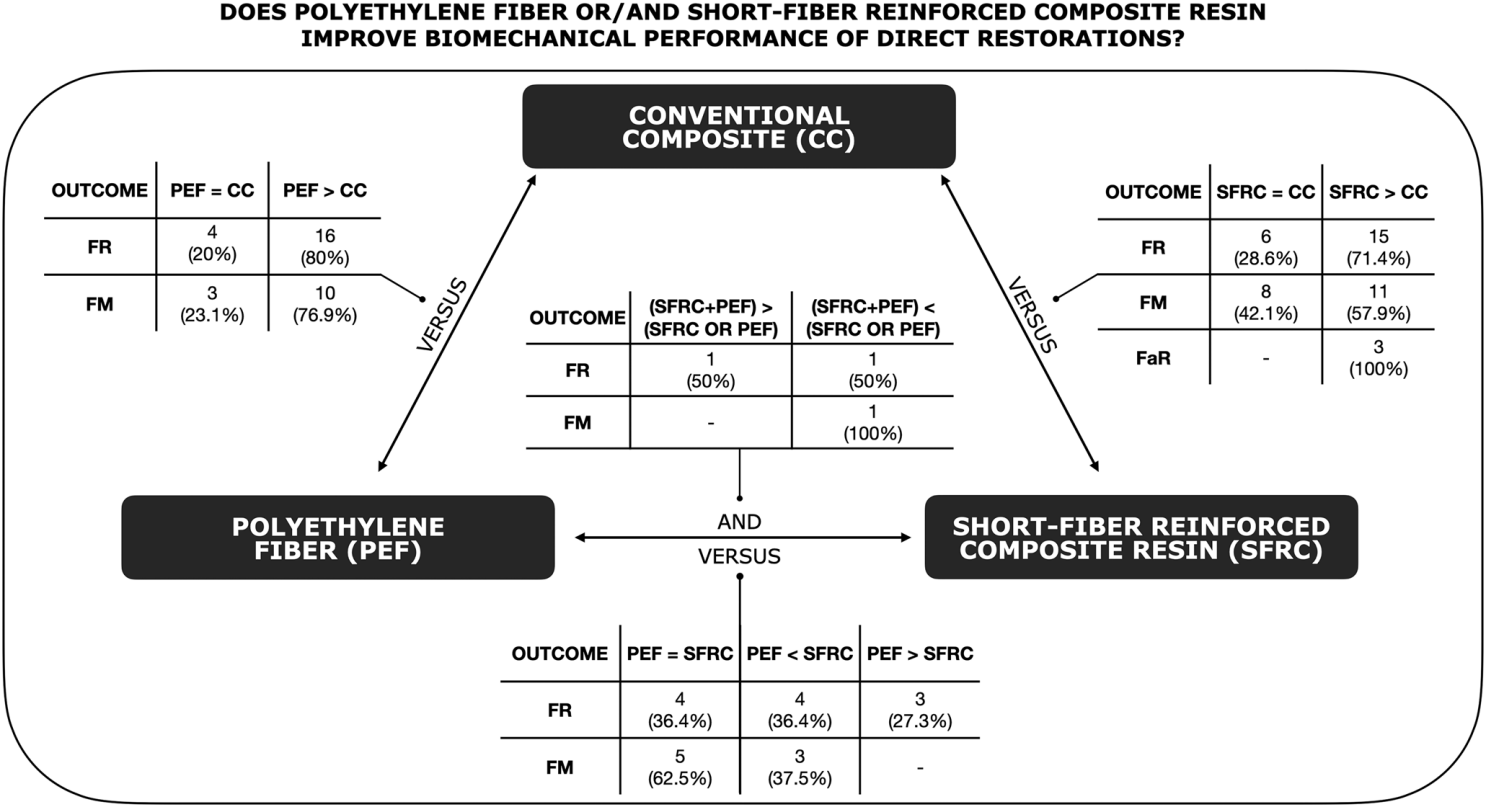
Schematic representation of the results of the included studies. FaR, fatigue resistance; FR, fracture resistance; FM, failure mode.

In order to assess the biomechanical performance, fracture resistance was evaluated in all studies except for two [27, 28], and failure mode was examined in 43 papers [12, 17, 19, 20, 21, 22, 23, 24, 25, 27, 28, 31, 32, 33, 35, 36, 37, 38, 39, 40, 41, 43, 44, 46, 47, 49, 50, 53, 54, 56, 57, 58, 61, 62, 63, 64, 65, 66, 68, 69, 70, 72, 73]. Thirty‐two (32) publications used aging methods to assess long‐term properties of the materials [12, 17, 18, 20, 21, 23, 25, 27, 28, 30, 31, 32, 33, 34, 35, 38, 39, 43, 44, 49, 50, 51, 52, 53, 60, 62, 63, 67, 69, 70, 71, 73]. A total of 24 studies appraised the SFRC application [17, 18, 19, 20, 21, 22, 23, 24, 25, 26, 27, 28, 29, 30, 31, 32, 33, 34, 35, 36, 37, 38, 39, 40], followed by polyethylene fiber, which was evaluated in 20 papers [12, 41, 42, 43, 44, 45, 46, 47, 48, 49, 50, 51, 52, 53, 54, 55, 56, 57, 58, 59], and both materials were tested and compared in 14 publications [60, 61, 62, 63, 64, 65, 66, 67, 68, 69, 70, 71, 72, 73] (Figure 2). The application techniques varied across the different studies. The angle at which the force was applied ranged from 0° to 135°. The control groups varied between intact [12, 17, 18, 19, 22, 23, 26, 28, 29, 30, 31, 32, 33, 37, 39, 40, 41, 42, 43, 44, 45, 46, 47, 48, 50, 51, 52, 54, 55, 56, 57, 58, 59, 65, 68, 69, 70, 71], unrestored [17, 18, 23, 29, 31, 37, 39, 42, 43, 46, 48, 50, 51, 52, 56, 57, 58, 59, 69, 70, 71] and restored teeth with CR [20, 25, 27, 34, 36, 38, 49, 60, 62, 64, 66, 67] or SFRC [36, 63].

## 8 Discussion

The rehabilitation of severely compromised teeth remains a significant challenge in daily clinical practice, with numerous factors to consider when extensive structural loss occurs. One of the most important factors to assess a tooth's prognosis is the presence or absence of marginal ridges. Plotino et al. [74] reported that for maxillary premolars, the loss of one marginal ridge leads to a decrease of 46% in tooth rigidity and fracture resistance, which further decreases to 63% when both ridges are lost. In contrast, when both marginal ridges are maintained, in a class I cavity, the decrease in stiffness and fracture resistance is only 20% [75]. In addition, endodontic treatment meaningfully contributes to the decline in fracture resistance, primarily due to the structural loss caused by carious lesions removal, access cavity preparation, mechanical canal preparation, dentin's water content reduction, and altered proprioception [3, 57, 76, 77, 78]. These factors should be considered when interpreting literature results, given the wide variety of cavity configurations performed across studies. Notably, among the analyzed studies, two marginal ridges were removed in 81% of the publications including posterior teeth, and endodontic treatment was performed in 74%.

Throughout the included publications, biomechanical performance was mainly assessed by determining fracture resistance. Therefore, the angle at which the force was applied is a critical factor to consider, as it highly varies across studies. The majority of papers (42) considered a 0° angle [12, 18, 21, 22, 23, 24, 26, 27, 28, 29, 30, 33, 35, 36, 37, 38, 40, 41, 43, 44, 47, 48, 49, 50, 52, 53, 55, 56, 58, 59, 60, 62, 63, 64, 66, 67, 68, 69, 70, 71, 72, 73], followed by a 45° angle in eight studies [17, 38, 42, 45, 46, 51, 54, 57], a 30° angle in four [19, 20, 61, 65], a 13.5° angle in one [34] and a 135° angle in another study [28]. Barreto et al. [38] compared the two most commonly used angles, concluding that fractures were mostly favorable when tests were conducted at 0°, whereas the inverse was observed when tests were performed at 45°.

Composite resins (CRs) have become the primary choice for direct restorations, due to their increasing customization [79]. Nevertheless, their use in large rehabilitations still presents challenges, such as achieving the ideal shape, contour, and occlusal anatomy [20]. Additionally, a momentous disadvantage of CRs is polymerization shrinkage, which induces stress at the interface between the restoration and the tooth substrate [20, 43, 80]. If the interfacial bond strength is exceeded, either adhesive failure or tooth fracture occurs [39, 43]. Furthermore, there is a tendency for cuspal deflection, enamel microfractures, marginal leakage, and postoperative sensitivity [81]. Additionally, the inadequate fracture toughness of these materials compared to dentin should also be pondered [82].

With the aim of reducing polymerization stress and achieving better mechanical properties, incremental layering techniques have been recommended, with horizontal or oblique increments applied at a maximum thickness of 2 mm [24, 83, 84]. These techniques reduce polymerization shrinkage at the restoration interface, consequently decreasing cuspal strain [85, 86]. However, there remains no consensus regarding the optimal technique [40].

Fiber reinforcement of direct restorations has been studied to address the shortcomings of CRs. SFRCs were developed to emulate the fibrous structure of dentin, attempting to replicate its mechanical properties [87]. EverX Posterior, consisting of short E‐glass fibers and barium glass particles (0.6–1.5 mm), is the main SFRC evaluated in literature [88]. These components are arbitrarily positioned within a resin matrix that contains conventional methacrylate monomers, such as bis‐GMA and TEGDMA, along with polymethyl methacrylate, forming a semi‐interpenetrating polymer network (semi‐IPN net: poly(methylmethacrylate)‐inter‐net‐poly(bis‐glycidyl‐A‐dimethacrylate)) that was alleged to offer good bonding and wetting to the tooth substrate [89]. Due to its composition, much higher flexural modulus and fracture toughness (12.6 GPa and 2.6 MPa $m^{1/2}$, respectively) distinguish it from previously existing materials [20]. Subsequently, EverX Flow was developed to address some clinical deficiencies of EverX Posterior, namely the difficult handling due to high viscosity and limited esthetics caused by the high translucency [20]. Nevertheless, the initial concern regarding polymerization shrinkage in nonreinforced CRs remains [90].

Delving into the assessment of the biomechanical performance of SFRCs, all studies included a nonreinforced CR group for comparison. The three parameters used to evaluate the performance of these materials were fracture resistance, assessed in 21 studies [17, 18, 19, 20, 21, 22, 23, 24, 26, 29, 30, 31, 32, 33, 34, 35, 36, 37, 38, 39, 40], failure mode in 19 [17, 19, 20, 21, 22, 23, 24, 25, 27, 28, 31, 32, 33, 35, 36, 37, 38, 39, 40], and fatigue resistance in 3 [25, 27, 28]. Pertaining to fracture resistance, the results either favored the application of SFRCs [17, 18, 19, 20, 21, 23, 24, 25, 26, 29, 30, 31, 33, 34, 36, 37] or showed no statistically significant differences between the two groups [22, 32, 35, 38, 39, 40]. A study by Bijelic‐Donova et al. [33] stood out for presenting higher fracture resistance values for teeth without endodontic treatment, restored with EverX Posterior, compared to the intact group, using an oblique incremental technique for dentin and CR for enamel. Two other articles achieved similar fracture resistance between intact teeth and SFRC restorations with incremental techniques [31, 37]. When the fatigue resistance parameter was evaluated, SFRC groups exhibited superior results in all studies [25, 27, 28].

Fiber‐reinforced composites present an anisotropic behavior, owing to the fiber distribution across the three planes. This characteristic has been associated with improved control of polymerization shrinkage and prevention of crack propagation [10, 11, 91]. Regarding the latter, Molnár et al. [27] observed a deflection of the fracture line upon contact with the SFRC. Nevertheless, this study also demonstrated that the failure mode of the teeth restored with SFRCs was still predominantly catastrophic, in resemblance to what was observed in the remaining groups [27]. Conversely, 11 studies included in this review reported that the use of SFRCs was beneficial [17, 19, 20, 21, 23, 24, 31, 33, 35, 36, 37], while eight studies argued that the application of these materials did not influence failure mode [22, 25, 27, 28, 32, 38, 39, 40].

Even though the application technique plays a pivotal role in the performance of reinforced composites, significant heterogeneity is reported among studies. Three different techniques were evaluated in the included studies for combining SFRCs and CRs, as well as three distinct SFRC applications. A bulk fill technique was applied in eight studies, with six reporting an increase in fracture resistance [17, 18, 19, 24, 28, 34], whereas the remaining studies did not observe this superiority over CR [38, 39]. It is noteworthy that no studies compared layering techniques to bulk fill. Furthermore, a different study compared the oblique and horizontal layering techniques using 2 mm thick increments of EverX Posterior [40]. The oblique technique demonstrated a tendency towards higher fracture resistance, and restorable fractures were observed exclusively in this group. Two publications described another technique, consisting of luting an individually made fiber‐reinforced post (Everstick Post, GC Europe N.V., Leuven, Belgium), followed by a core build‐up with flowable SFRC (EverX Flow, GC Europe N.V., Leuven, Belgium) [25, 28].

Considering the included studies that focused on class II restorations, 29% of the cases involved the entire outer layer of the restoration being made with conventional resin, in 61% only the occlusal surface was restored with CR, and in 10% of the publications the restoration was entirely made with SFRC.

As an alternative to fiber‐reinforced composites, polyethylene fibers were introduced, with Ribbond being the trademark material used in nearly all studies. This material is recognized for its leno weave structure, which is considered sufficiently open to permit efficient wetting and infusion of the resin, while also facilitating easier handling and adaptation to the teeth contours [47, 58]. The incorporation of polyethylene fibers within the restoration serves to optimize the distribution of stress over a wider area, provide multiple loading paths, and enhance the overall performance of restorations [4]. Another distinctive feature of Ribbond is its cold gas plasma treatment, which chemically alters the material from hydrophobic to hydrophilic, thereby improving resin impregnation [58]. To evaluate the biomechanical performance of this material, 20 studies measured fracture resistance [12, 41, 42, 43, 44, 45, 46, 47, 48, 49, 50, 51, 52, 53, 54, 55, 56, 57, 58, 59] and 13 evaluated failure mode [12, 41, 43, 44, 46, 47, 49, 50, 53, 54, 56, 57, 58]. Regarding fracture resistance, an increase was predominantly reported in 84,2% of the studies, while 76,9% described an improvement in the failure mode.

Fiber application technique emerges as one of the most variable factors within the cluster of studies considered, potentially influencing the obtained results. Among the selected publications, six different techniques were tested. For posterior teeth, the two most used techniques were the placement of fibers into the buccal, lingual, and pulpal cavity walls [43, 44, 47, 48, 49, 50, 51, 53, 56, 59] or the creation of a bucco‐lingual groove on the occlusal surface of the restoration in which the fiber was embedded [42, 46, 49, 52, 57, 58]. Rahman et al. [49] compared these techniques both individually and combined, concluding that the application of fiber in the occlusal portion of the restoration resulted in higher fracture resistance than placement at the base of the cavity. However, the base application led to a greater number of favorable fractures. The application technique within an occlusal groove can present a clinical problem, as substantial finishing procedures may expose the fiber, potentially leading to its degradation upon exposure in the oral cavity. Predictably, the combination of both techniques yielded the highest fracture toughness value [49]. The three other variations included the application of fibers into the buccal, lingual, and pulpal walls and, as well as between the two layers of composite [44]; a combination of buccal, lingual, and pulpal wall application with bucco‐lingual placement in an occlusal groove [49]; the application buccal to lingual at the marginal ridge area [41, 55] and a combination of the latter with the application on the pulpal floor [41]. For central incisors, two studies applied Ribbond in a palatal groove, showing that failure mode and fracture resistance significantly improved compared to CRs, achieving values comparable to those of intact teeth, thus appearing promising for reinforcing anterior teeth restorations [45, 54].

Delving into understanding which type of fiber yields the best results and whether their combination is beneficial, 14 studies addressed this topic without reaching consensus [60, 61, 62, 63, 64, 65, 66, 67, 68, 69, 70, 71, 72, 73]. Ozsevik et al. [71], Garlapati et al. [70], Tsertsidou et al. [62] and Mossad Hassan Negm et al. [60] agreed that EverX exhibited superior fracture resistance compared to polyethylene and CRs, additionally presenting a more favorable [62] or similar failure mode [70]. Furthermore, Ozsevik et al. [71] reported fracture resistance values comparable to those of intact teeth, with fiber‐reinforced resins, ensuring that proximal walls and a 2 mm occlusal layer were restored with CR. Conversely, Abdulamir et al. [64], Sáry et al. [68] and Volom et al. [63] concluded that the fracture resistance values of polyethylene fiber were higher than those of fiber‐reinforced resins, although the failure mode was similar [63, 64] or even worse [68] than that of tested SFRCs. Notwithstanding Sáry et al. [68] reporting predominantly unfavorable failure modes, higher fracture resistance was observed for polyethylene fibers compared to intact teeth. The polyethylene fiber was applied according to the transcoronal splint technique, where the fiber is placed through penetrations made in the buccal and lingual walls into the grooves prepared on the external coronal surfaces, connecting the opposing walls like a tightrope—a technique not previously mentioned in the literature.

Furthermore, four studies found no statistically significant differences in the fracture resistance of polyethylene and fiber‐reinforced composite resins [66, 69, 72, 73]. Regarding the failure mode, two studies found no variation [66, 69]; one favored the use of polyethylene fiber [73] and one the use of fiber‐reinforced resin [72]. In three of these studies, the fiber was applied circumferentially to the axial walls of the cavity, a technique not mentioned in previous studies and not proven to be superior to the described alternatives [61, 64, 68].

The effects of the simultaneous application of polyethylene fiber and SFRCs in restorations exhibit inconsistencies in the available literature. Soto‐Cadena et al. [65] reported an improvement in fracture resistance, although the failure mode was more favorable when the materials were used separately. In contrast, Albar et al. [67] concluded that applying these materials separately resulted in superior fracture resistance. Additionally, Ramírez‐Gómez et al. [61] investigated various application techniques of polyethylene fibers in combination with fiber‐reinforced resins, finding all techniques beneficial for the failure mode. However, they noted that the application of the fibers in more than one direction (bucco‐lingual and mesio‐distal) was associated with decreased fracture resistance compared to unidirectional fibers applied from mesial to distal, which yielded the highest fracture resistance [61].

The conspicuous lack of consensus among the reviewed studies unequivocally precludes the formulation of definitive conclusions concerning the comparative analysis and combined use of these restorative materials. Several significant inconsistencies were identified across key parameters, including sample selection. Molars, premolars, and incisors were used across studies. Considering their distinct biomechanical characteristics, meaningful comparisons between these groups are impractical. Disparities were also evident in cavity preparation techniques. Two main approaches were identified: standardizing the isthmus dimensions or the thickness of the remaining walls. In the first, the amount of restorative material applied may be consistent across the groups, but the thickness of the remaining tooth structure can vary significantly within and between the groups. In contrast, the latter technique maintains consistent wall thickness, independent of the specimen's dimensions or material volume. Another critical inconsistency was related to whether or not root canal treatment had been performed. This is a key variable as such procedure increases the tooth's fragility, primarily due to the loss of structure. Furthermore, there was no agreement on the application techniques, regardless of the used restorative material, which also jeopardizes outcomes' comparability across studies. Differences in testing methods, particularly in fracture resistance tests, further contributed to the results heterogeneity. Variations in the force application angles across studies likely influenced the results, limiting the ability to generalize findings. In addition, to improve the clinical relevance of future research, it would be advantageous to simulate more accurately the conditions that teeth endure in the oral cavity. Since teeth are subjected primarily to cyclic loads rather than constant forces, fatigue resistance testing may offer more meaningful insights. Moreover, the omission of sample size calculations in many studies represents a major limitation, as it undermines the statistical power and reliability of their conclusions. To address these issues, future studies should adopt standardized methodologies and rigorously defined parameters to produce more reliable and clinically relevant results.

## 9 Conclusion

According to this scoping review, the following conclusions are drawn:

- Based on the considerable disparity between the parameters assessed across the studies—involving tooth type, cavity configuration, root canal treatment, restorative material application technique, and testing methodology—it is recommended to establish standardized guidelines for future research. Such standardization will enhance the comparability of results and facilitate the translation of findings into clinical practice.
- The individual application of fiber‐reinforced composite resins and polyethylene fibers demonstrates promising potential in direct restorations. No adverse biomechanical outcomes were reported, with findings either supporting their use or indicating no significant difference. Nevertheless, the results are ambiguous and insufficient to directly compare both materials or assess the effects of their combined use. Further research is warranted to consolidate these findings and explore long‐term outcomes.

## Disclosure

The authors have nothing to report.

## Conflicts of Interest

The authors declare no conflicts of interest.

## Data Availability

The data that support the findings of this study are available from the corresponding author upon reasonable request.
